# Interaction between the oculomotor and postural systems during a dual-task: Compensatory reductions in head sway following visually-induced postural perturbations promote the production of accurate double-step saccades in standing human adults

**DOI:** 10.1371/journal.pone.0173678

**Published:** 2017-03-15

**Authors:** Mathieu Boulanger, Guillaume Giraudet, Jocelyn Faubert

**Affiliations:** 1 École d’Optométrie, Université de Montréal, Montreal, Quebec, Canada; 2 Jewish Rehabilitation Hospital, McGill University, Laval, Quebec, Canada; Ludwig-Maximilians-Universitat Munchen, GERMANY

## Abstract

Humans routinely scan their environment for useful information using saccadic eye movements and/or coordinated movements of the eyes and other body segments such the head and the torso. Most previous eye movement studies were conducted with seated subject and showed that single saccades and sequences of saccades (e.g. double-step saccades) made to briefly flashed stimuli were equally accurate and precise. As one can easily appreciate, most gaze shifts performed daily by a given person are not produced from a seated position, but rather from a standing position either as subjects perform an action from an upright stance or as they walk from one place to another. In the experiments presented here, we developed a new dual-task paradigm in order to study the interaction between the gaze control system and the postural system. Healthy adults (n = 12) were required to both maintain balance and produce accurate single-step and double-step eye saccades from a standing position. Visually-induced changes in head sway were evoked using wide-field background stimuli that either moved in the mediolateral direction or in the anteroposterior direction. We found that, as in the seated condition, single- and double-step saccades were very precise and accurate when made from a standing position, but that a tighter control of head sway was necessary in the more complex double-step saccades condition for equivalent results to be obtained. Our perturbation results support the “common goal” hypothesis that state that if necessary, as was the case during the more complex oculomotor task, context-dependent modulations of the postural system can be triggered to reduced instability and therefore support the accomplishment of a suprapostural goal.

## Introduction

In everyday life situations, context-dependent regulation of posture is often required to produce accurate goal-directed behaviors while standing. Reaching for an object and shifting gaze to different objects of interest for further scrutinization are but two examples of so-called suprapostural tasks that may necessitate such regulation. Research on the control of posture during goal-directed eye movements in healthy adults has produced variable results and a consensus has yet to be reached. Though different measures of posture were used across studies to assess postural changes during saccadic eye movements (e.g. head or torso sway [1–5] and body sway [2, 3, 5–19], the results can nevertheless be grouped into mainly two categories. Some found that saccadic eye movements have no effect on posture [2, 3, 12, 14, 19], while others reported decreases in sway [1, 4, 5, 9, 11–18, 20].

Whether one postulates that postural control and the performance of a concomitant suprapostural task, such as eye movements, tap into a common and limited pool of central resources [21, 22] or that instead the two systems collaborate to achieve a higher-level, common goal [23, 24] in our opinion can best be determined with proper quantification of eye movement accuracy and posture, and by varying the complexity of the tasks. Here we tested the validity of those two hypotheses. We report measurements of eye movement accuracy and modulations of head sway in standing human subjects as they performed oculomotor tasks of varying complexities that required either to maintain fixation on a stationary target, to perform single-step saccades or to generate sequences of double-step saccades. In many of the experiments, balance was also challenged using visually-induced postural perturbations. This new dual-task paradigm therefore required to concomitantly maintain balance and to accurately fixate gaze targets.

To our knowledge, we are the first to report on the generation of double-step saccades by human subjects from a standing position. It has been shown repeatedly in seated subjects that double-step saccades are accurate [25–27], like single-step saccades are, but we currently don’t know if it’s also the case when they are made from an upright stance. More processing steps have to be performed in order to produce double-step saccades, compared to single-step saccades. One such operation is usually referred to as spatial updating or target remapping [28]. Target remapping is crucial during double-step saccades because the retinal coordinates of the second target at the end of the first saccade are different from those computed following its brief presentation during the fixation period that preceded the saccade sequence. In other words, for the second saccade to be accurate, the endpoint of the first saccade of the sequence has to be taken into account when producing the motor command that will bring the second eye movement on target. No such computations are necessary during single-step saccades. For this reason, we classified the double-step saccades performed by our subjects as more complex than single-step saccades.

If the oculomotor and postural systems compete for limited resources, one can hypothesize at least two scenarios. In the first one, a trade-off in performance would be observed, i.e. improvement on one task would lead to deterioration on the other task. In the second scenario, performance on both tasks would deteriorate since attention is divided to complete the two tasks concomitantly. On the other hand, if the two systems instead collaborate to attain a common goal, changes in performance will be observed only when necessary. In the context of our experiments, as previously hypothesized by Stoffregen et al. [4], this would mean that accurately fixating the visual targets would be the primary goal and that whether adaptive postural behaviors are observed or not would depend on how detrimental to the overall goal it would be not to adapt. Following this logic, reducing body sway would not serve the sole purpose of maintaining balance, but instead would promote the production of accurate eye movements by providing a more stable base of support.

Our working hypothesis was that the endpoint accuracy of both single- and double- step saccades would be preserved despite experimentally-induced postural perturbations. Since the horizontal component of the required saccades was much larger than the vertical one during our experiments, we further hypothesized that tighter control of posture in the form of a reduction in mediolateral (ML) head sway would be observed in order for saccades to remain accurate, and that this reduction in head sway would be more prominent in the double-step saccade condition. We hypothesized a reduction mainly of ML sway based on the so-called minimal intervention principle, which states that larger variance is usually found in task-irrelevant directions [29–31]. In the context of our research, this would mean that reduction in sway should be expected mainly, if not only, along the principal movement axis of the eye saccade. Concerning the sway modulation primarily during the double-step saccades, our reasoning was that a stricter control of balance, in our case a more stable upper body, would be necessary to produce accurate double-step saccades due in part to their more complex nature.

## Materials and methods

### Subjects

Twelve healthy subjects (3 males) participated in the experiments. Their ages ranged from 22 to 27. All subjects were completely naive regarding the specific objectives of the study, and all had normal or corrected-to-normal vision. The experimental approach was approved by the Research Ethics Board of the Université de Montréal. Subjects all gave written informed consent.

### Apparatus

All experiments were conducted in the dark (background luminance = 3.2 cd/m^2^). Stimuli were back-projected onto a large opaque screen with an inclination of 17° (with respect to the earth’s horizontal plane). Given the inclination of the screen, the portion of the screen closest to- and farthest from- the subject covered a visual angle in the horizontal plane of 145° and 45°, respectively (screen resolution: 1024 × 768 pixels, frame rate 120 Hz). In all experiments reported below, the eye-to-screen distance when the subject was looking straight ahead was ~96 cm.

Subjects stood with both feet together in front of the screen with their upper torso positioned a few centimeters from the bottom part of the screen and with the body midline aligned with the center of the screen. Each subject’s vertical position was adjusted such that an approximate constant eye-to-screen distance (see above) was obtained across trials and across subjects as they looked straight ahead at a red fixation point projected at eye level in the upper part of the screen. Subjects wore a fitted and very snug, porous helmet-headband to which an eyetracker was attached (Eyelink 1000, SR Research, Ontario, Canada). Movements of the right eye were measured with this video system, whose algorithm tracked the center of the pupil. The eye tracker cameras were positioned close to the subject’s face, just below the eye at an angle of ~45°. The eye camera sampled eye position data at 500 Hz. Before each experiment, a first eye-in-head calibration routine was performed by having subjects fixate each point of a 3x3, 9-point calibration grid. This initial calibration was done from a seating position and targets were presented on the PC screen provided by the eyetracker manufacturer. The subject-to-screen distance during this calibration was 96 cm. A second calibration was performed with the standing subject facing the large screen used during the experiments. The eye-to-screen distance during this second calibration routine was also 96 cm. The subject maintained its head in the straight ahead direction by aligning the beam of a laser fixed at the center of the eyetracker headband with a long vertical blue bar back-projected at the center of the screen. Visual targets were back-projected onto the screen at known spatial positions. The associated eye-in-head positions were related offline to the known spatial location of each point and converted to degrees of angle.

Head-in-space position was recorded in 3-D using 7 infrared cameras (Optitrack, NaturalPoints, Oregon, U.S.A.) positioned on the ceiling all around the subject that detected the position of three markers secured to a lightweight tripod, itself attached to the eyetracker headband. The head recording system was calibrated by having subjects align the light-spot from the head-attached laser beam with an array of white circles back-projected sequentially onto the translucent. The head recording system sampled head position data at 100 Hz. Head signals were related offline to the known spatial location of each point and were converted to degrees of angle. Subjects were not required to generate rotational movements of the head during our experiments (see below), but we nevertheless assessed whether the subject’s head was pointing forward as requested and did not rotate in concert with the saccadic eye movements.

The onset and offset of all events and data acquisition (sampled at 1 KHz) and storage were controlled using an in-house program. All stimuli were created using C++ and OpenGL 3.0. Eye-tracker and head angular displacements output signals were all acceptably linear within a range of about ±40°.

### Experimental tasks

#### Double-step saccade experiments

**Fig 1:**
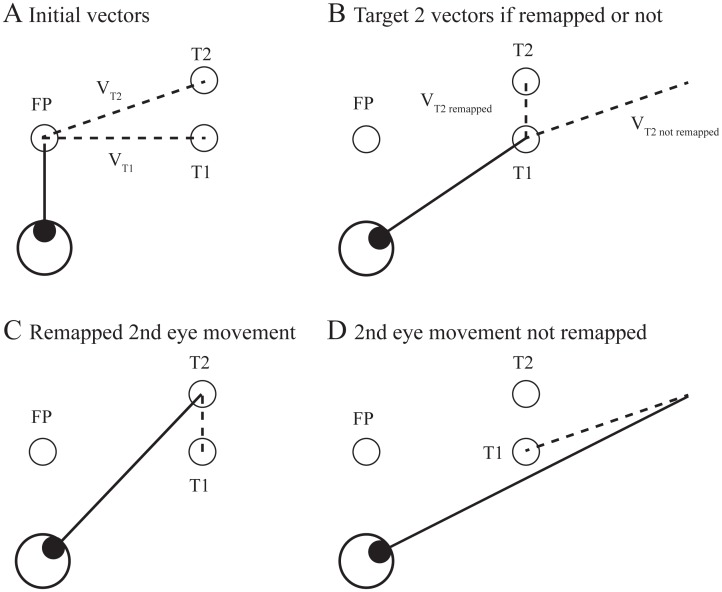
Double-step saccade task. (A) Saccade vectors encoded during initial fixation period for both the first (T1) and second (T2) targets of the sequence. Both targets are presented during the fixation period and are extinguished before the onset of the first saccade. (B) Saccade vector of the second saccade of the sequence at the end of the first saccade of the sequence. (C) Successful acquisition of T2 following remapping of V_T2_. (D) Large second saccade errors observed if no remapping is performed. FP = fixation point. T1 = target 1. T2 = target 2. V_T1_ = vector of first saccade.V_T2_ = vector of second saccade. Solid line = gaze direction. Dashed lines = encoded saccade vectors.

In the first set of experiments, subjects were required to produce, from an upright stance, double-step saccadic eye movements from an initial fixation point (FP duration = 1000–1500 ms) to the remembered location of two briefly (Target 1 duration = 100 ms; Target 2 duration = 80 ms) and sequentially presented visual targets and maintain balance despite the perturbing effect of the peripheral stimulus. As mentioned in the Introduction, target remapping is of great importance during the production of accurate double-step saccades. This is so because the retinal vector of the second target encoded during the initial fixation period (Fig 1A) differs greatly from that encoded after the intervening first saccade (Fig 1B). Fig 1C and 1D show the endpoint of the second saccade during a hypothetical double-step saccade sequence that was properly remapped (Fig 1C) and one that was not remapped as it should following the first saccade of the sequence.

**Fig 2:**
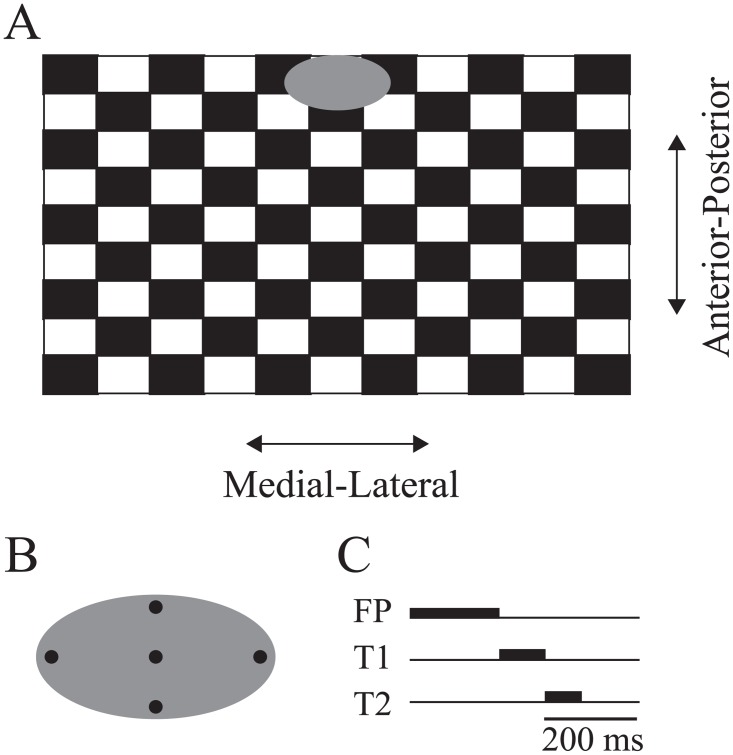
Visual stimuli used in the experiments. (A) Illustration of the checkerboard pattern that moved sinusoidally either in the anterior-posterior or medial-lateral direction and of the grey oval patch inside which gaze targets were projected. The oval patch itself did not move, so the checkerboard pattern moved “behind” the oval. (B) Position of the gaze targets presented inside the oval patch. Note that all targets were flashed for short durations and that two targets were never presented simultaneously. (C) Timing of stimuli presentation during a typical double-step saccade experiment. All saccade targets were extinguished before the onset of the 1st eye saccade. The same targets positions were used in the single-step saccade condition and in the fixation condition.

The sequence of saccade targets presentation is shown in Fig 2C, while Fig 2B shows the positions of the visual targets that were used during the experiment. The initial red fixation point and the subsequent two green saccade targets were chosen among those 5 possibilities at random prior to each “eye movement trials”, with no repetition of the same dot during a given saccade trial. The angular distance from the central target to: (1) the horizontal targets were 6.25°; (2) the bottom target was 1.75°; (3) the top target was 1.5°. Vertical offsets (top and bottom targets) were used to discourage anticipation and therefore add a random element to our task. Only horizontal eye movements were analyzed in this study. All saccade targets were projected inside a grey oval (23x20 cm or 14x12°) located at the top of the screen and centered with respect to the subject’s body midline. The rest of the screen was filled with a black and white checkerboard pattern that could either remain static throughout a trial or move either in the anterior-posterior (i.e. front-back) or the mediolateral (i.e. side to side) direction (Fig 2A). Note that in Fig 2A, the screen inclination is not depicted. Given the screen inclination (see above), what appears to be up-down motion in the figure is actually anterior-posterior motion. The luminance of the black and white squares were 0.63 cd/m^2^ and 24.4 cd/m^2^, respectively, and the spatial resolution of the pattern was 100 pixels/cm^2^. In the *dynamic conditions*, sinusoidal translational motion of 15° was applied to the checkerboard at an oscillating frequency 0.25 Hz either in the anteroposterior (AP) direction or in the mediolateral (ML) direction. The direction of motion of the checkerboard stimulus was chosen at random from one trial to the next. In the *static condition*, the checkerboard was present but remained immobile throughout the trial. One trial with a given checkerboard lasted 32 seconds and 12 sequences of double-step targets were projected on the screen during that period.

#### Single-step saccade experiments

In a second set of experiments, subjects had to make single-step saccades also from a standing position and under the same *static* and *dynamic* checkerboard conditions (see above). The only difference was that following the extinction of the red FP, only one green saccade target was briefly flashed (duration: 80 ms) and as such only one eye movement had to be produced at a time. As described above, one trial with a given checkerboard lasted 32 seconds and 24 single-step targets were projected on the screen during that period.

#### Fixation experiments

In yet another set of experiments, standing subjects were asked to keep their gaze fixed on a red FP presented on the screen inside the gray oval for the duration of the trial (i.e. 32 seconds). As was the case during the eye saccade experiments the position of the FP was randomly chosen among the five different possibilities. Importantly, in previous experiments body sway measured during saccadic eye movements were compared with a condition in which subjects fixated a central static visual stimulus, leaving open the possibility that differences across conditions could be due to a simple static eye position effect. Here we controlled for this possible confound by using the peripheral saccade targets as static fixation stimuli during the fixation condition. We emphasize this important difference in methodology because it has been shown that static eye-in-orbit deviations and low amplitude mechanical vibrations to the extraocular muscles of the eye both lead to changes in posture [32–35]. It is interesting to note also that static eye-in-head deviation has also been shown to induce deviations in walking direction [36]. Fixation experiments were conducted under both *static* and *dynamic* checkerboard conditions.

### Experimental procedure

As alluded to earlier, all experiments were conducted from a standing position, with the feet together. The sequence in which the tasks were administered was the same for all subjects. After completing the eye and head calibration routines described in the previous section, each subject first completed 10 trials during which they performed 12 sequences of double-step saccades with the checkerboard moving randomly in one of the two directions (*dynamic* condition). The duration of each trial was 32 seconds. Double-step saccade trials with the static checkerboard were then tested in a second block of 10 trials (*static* condition). Subjects took a short 3-minute break between the *dynamic* and *static* blocs of trials. During the same session, and following a 5-minute break during which subjects sat on a chair with their eyes closed, subjects then performed another set of 10 trials of 32 seconds during which they performed single-step saccades in the presence of the same peripheral checkerboard pattern (*dynamic* and *static* conditions). Again, subjects were allowed a short 3-minute break between the dynamic and static conditions. Finally, subjects were asked to come back for a second session during which they participated in the fixation experiment (10 trials of 32 seconds) with the same checkerboard pattern (both dynamic and static conditions also tested). Eye and head movements were recorded during the fixation experiments and the same eye and head calibration routines described above was done prior to running these experiments.

During all experiments, subjects were specifically instructed to concurrently perform accurate saccades from a standing position and maintain a stable upright stance. They were required to place equal emphasis on each of these two experimental requirements.

### Data analysis

Eye and head position signals were imported to Matlab (Mathworks) for further off-line analysis. All signals were low-pass filtered (80 Hz) using a digital filter with zero-phase lag in Matlab (filtfilt.m).

#### Eye position analyses

Though overt rotational head movements are more likely to accompany gaze shifts larger than about 15 degrees, it should not be assumed that small head rotations won’t occur when smaller gaze shifts are generated [37–39]. Moreover, it was reported using a variety of techniques that neck muscle modulations (head-turning synergies) are observed in many species, including humans, during small amplitude (and medium) gaze shifts (≈5–20°) and eye-in-head deviations smaller than 15 degrees [40–50]; for a discussion see Corneil and Munoz [51]. Before calculating eye displacements, we therefore made sure that subjects did not concomitantly rotate their head as they gazed at the different visual targets and therefore kept the head pointing forward at all time. In seated human subjects, angular head displacements are seldom produced during small saccades such as the ones generated by our subjects [38, 52], but we nevertheless made sure that the results presented below from standing subjects would not be confounded by this. For this reason, we ran extensive analyses of head rotation and found no clear evidence that subjects rotated their head during any of the tasks (see S1 File). Schärli et al. [2] also reported similar head rotation results in a group of young adults performing gaze shifts from a standing position. Gaze movement data will therefore be presented in the form of eye displacements with respect to the center of the orbit (i.e. eye-in-head). The distributions of absolute eye displacements (i.e. unsigned) made to the left and right visual targets did not differ (t-test, p>0.05), so we pooled rightward and leftward saccade displacements. In the case of double-step saccades, we were interested in knowing whether the endpoint of the second saccade (see Introduction) would be affected across conditions so only those are presented here. All subjects easily maintained fixation during the fixation task (data not shown).

Horizontal eye-in-head position distributions were first generated in order to examine where gaze was directed with respect to the visual targets. This was done separately for each 32-second trial. In all oculomotor conditions, all trials and for all subjects three clear horizontal eye position distributions were observed, corresponding to the three positions of the horizontal saccade targets. We then confirmed the normality of all eye position distributions using the Kolmogorov-Smirnov test (p>0.05). The mean and standard deviation (std) of each distribution were then calculated. Mean values were used to indicate whether subjects on average made accurate saccades and the std values were used as a measure of precision. The reader is referred to Sommer and Wurtz (2004) [53] for a similar decomposition of eye movement distributions into separate measures of accuracy and precision. Mean angular eye position data was then converted to angular eye displacement by subtracting the eye position data obtained as subjects gazed at the two peripheral visual targets from that obtained while subjects gazed at the central visual target. We therefore ended up with an eye displacement value for saccades that were made to the leftward visual target and one eye displacement value for eye movements that were made to the rightward visual target. T-tests revealed no significant differences between rightward and leftward displacements so eye movements made to the two peripheral targets were pooled (p>0.05). Similarly, std values for movements made to the right visual target and those made to the left visual target did not differ which justified the pooling of those values as well.

For a given subject, we then pooled eye displacement and std data across trials, separately for each of the checkerboard conditions (mediolateral, anteroposterior, static) and for each oculomotor task (Fixation, Single-step saccade, Double-step saccade). For each condition, we also generated standard error of the mean values that were than used to plot error bars in the bar graphs presented below. Before pooling data across trials, individual trials were first plotted together and visual inspection confirmed that no outliers were present in the data before mean values were generated. Data points all lay within 1 standard deviation of the mean. Note also that learning effects were not observed which justified pooling across trials. This was verified by plotting mean and std values as a function of trial number and then generating linear regression slopes. None of the slopes differed from zero (t-test, p>0.05). For each condition, we then pooled mean and standard error data from all 12 subjects and generated grand mean and standard error values. Again, data from each subject was plotted together with the grand mean data from the 12 subjects to verify that no outliers were present in the dataset before running further statistical tests. Visual inspection of the plot of overlaid group and individual data again confirmed that all data points were within 1 std of the grand means (both for mean and standard error values).

#### Head sway analyses

Three measures we extracted from the head position data that were used to quantify head sway: front-back displacements of the head or 1-D anteroposterior (AP) head sway, side-to-side displacement of the head or 1-D mediolateral (ML) head sway and combined AP and ML head displacements or 2-D head sway. The velocity root mean square (vRMS) of each of the three measures was then computed and used to quantify postural fluctuations measured at the level of the head. In the following, increases in the fluctuations will be described as increases in instability. By squaring the velocities and taking the square root, we overcome the “directional” component of the signals and simultaneously compute the average velocity of the signal over an extensive recording period. vRMS allows us to simply quantify the “random walk-like” fluctuation patterns present in the sway signals. vRMS also has the advantage of providing a measure that reflects velocity changes at all frequencies, not only at the frequency directly associated with the movement of checkerboard stimulus. Recent studies in our laboratory used a similar measure [54–56].

### Statistical analyses

The data was analyzed using a within-subject design. Repeated-measures ANOVAs were used to investigate the effect of both oculomotor condition and background stimulus on head sway and also to study whether and how eye movement precision and accuracy were affected by the type of eye movements performed and the type of background stimulus used. When significant effects were present, post-hoc comparisons were done using the Bonferroni method. Additional details will be provided in the Results section when necessary. Statistical significance was determined using a P value of 0.05. All statistical analyses were done in SPSS.

## Results

In this study we investigated the interaction between the oculomotor and postural systems. We designed a new task in which subjects had to maintain balance and perform accurate saccades from a standing position in the presence of visually-induced postural perturbations. We will first present analyses of gaze accuracy.

### Saccade accuracy is preserved across conditions

**Fig 3:**
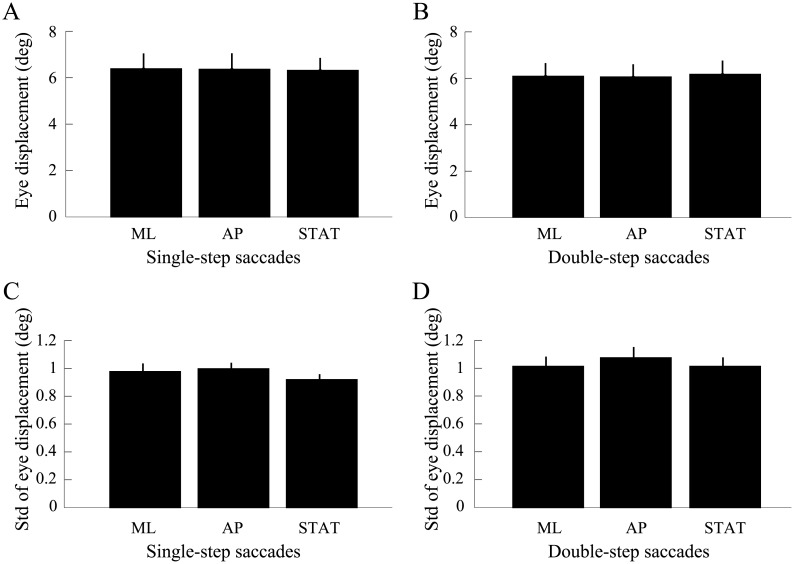
Eye movement results. Accuracy (A,B) and precision (C,D) of eye displacements as a function checkerboard stimulus movement in the single- step (A,C) and double-step (B,D) saccade conditions. Displacement data is plotted with respect to the central gaze target position. Data from all subjects and trials was pooled. Error bars = standard error of the mean. ML = mediolateral, AP = anteroposterior, STAT = static.

Fig 3 shows how horizontal eye displacements produced during the single- (Fig 3A and 3C) and double- (Fig 3B and 3D) step saccade tasks and under the different peripheral visual stimulus conditions (ML = mediolateral; AP = anteroposterior; STAT = static). Pooled data from all subjects is presented here. The small error bars attest to this. Mean eye displacement is shown in the top two panels (Fig 3A and 3B) and eye displacement variability is presented in the two bottom panels (Fig 3C and 3D). As is readily apparent from this figure, gaze shifts were equally accurate (Fig 3A and 3B) and precise (Fig 3C and 3D) under all conditions. Repeated-measures ANOVAs confirmed that eye movement precision did not differ across conditions (STIM: F_(2,22)_ = 1.92, p > 0.05; TASK: F_(1,11)_ = 2.65, p > 0.05), but a significant difference was found in terms of eye displacement as a function of the oculomotor task performed (TASK: F_(1,11)_ = 21.38, p < 0.01, η_p_^2^ = 0.66). No effect of stimulus was found (STIM: F_(2,22)_ = 0.13, p > 0.05) and no significant interaction was observed (F_(2,22)_ = 1.76, p > 0.05). The effect of task revealed that the single-step saccades terminated slightly more eccentric than double-step saccades when the dynamic peripheral stimuli were used. We calculated the difference in mean endpoints among those conditions in degrees of angle and found that it was very small, i.e. 0.3°, a difference that is in assuredly meaningless given that our system’s spatial resolution, as reported in the user manual, is 0.5°. The accuracy and precision of eye saccades therefore appear to be equivalently good in all conditions tested.

### Visually-induced mediolateral head sway decreases during double-step saccades

We showed in the previous section that gaze accuracy and precision did not differ across conditions. Postural data will now be presented. We start our exploration of postural behaviour by presenting a global measure of instability that combines both anteroposterior and mediolateral head sway into a single measure of posture disturbance (2-D vRMS).

**Fig 4:**
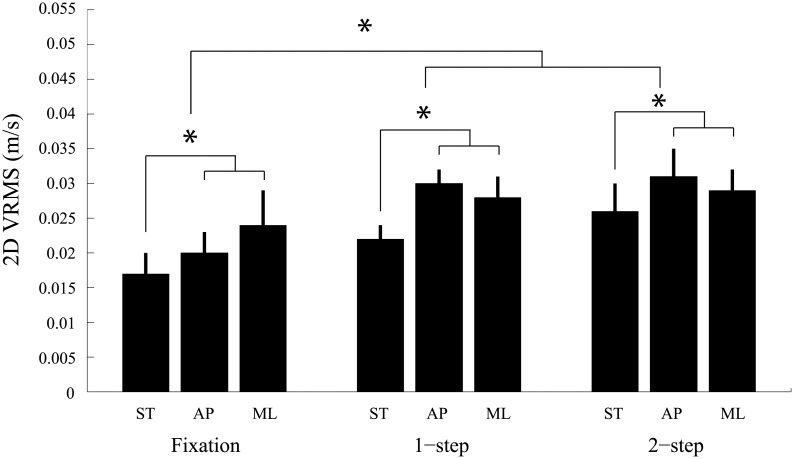
Mean 2-D velocity root mean square (vRMS) as a function of background stimulus (checkerboard conditions: ST = static, AP = anteroposterior, ML = mediolateral) and oculomotor task (fixation, 1-step saccade, 2-step saccade). Data from all subjects and trials were pooled. Error bars = standard error of the mean.* denotes a significant difference (p < 0.05).

Fig 4 shows the 2-D vRMS for all three oculomotor tasks and for all three peripheral visual stimulation conditions. Pooled data from all 12 subjects is shown. A repeated-measures ANOVA analysis with peripheral stimulus (STIM) and oculomotor task (TASK) as factors revealed a significant effect of both factors (STIM: F_(2,22)_ = 15.31, p < 0.001, η_p_^2^ = 0.58; TASK: F_(2,22)_ = 5.96, p < 0.01, η_p_^2^ = 0.35), but no significant interaction (F_(4,44)_ = 1.46, p = 0.23, η_p_^2^ = 0.12). Post-hoc analyses were performed using the Bonferroni method. The latter first revealed that more postural instability was observed using the dynamic peripheral stimuli then using the static stimulus (anteroposterior stimulus vs static stimulus: p < 0.01; mediolateral stimulus vs static stimulus: p < 0.05), but that equivalent increases of instability were found using either of the dynamic stimuli (anteroposterior stimulus vs mediolateral stimulus: p > 0.1). Post-hoc comparisons also showed that equivalent postural instability was observed during the single and double saccade task (single saccade vs double saccade: p > 0.1), but that more or marginally more instability was found when saccade tasks were contrasted with the fixation task (single saccade vs fixation: p = 0.067; double saccade vs fixation: p < 0.05). Though it failed to reach significance, when one compares the static visual condition across oculomotor tasks, it appears that the simple act of making a saccade increased head sway. To account for this trend, for the remainder of the analyses we normalized the postural data for each oculomotor task separately using data obtained with their respective static visual condition (dynamic/static x 100). We will therefore present % changes in head sway with respect to the static condition. Values larger than 100% mean that head sway was larger during the dynamic condition. With this normalization procedure we were able to use the static condition as a baseline against which the effects of visually-induced perturbations could be investigated in isolation.

**Fig 5:**
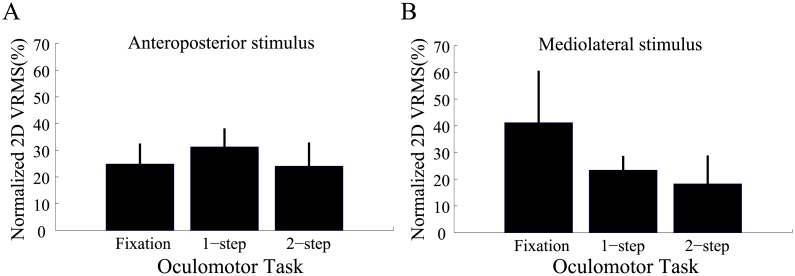
Mean normalized 2-D vRMS as a function of oculomotor task. Checkerboard stimulus moving in the (A) anteroposterior and (B) mediolateral directions. Data from all subjects and trials was pooled. Error bars = standard error of the mean.

Normalization applied to our 2-D postural data (Fig 5) revealed no significant effect of the oculomotor task on posture for both dynamic peripheral stimuli (anteroposterior stimulus: F_(2,22)_ = 0.24, p > 0.1; mediolateral stimulus: F_(2,22)_ = 0.76, p > 0.1). Though no significant differences were found across oculomotor tasks when subjects performed saccades in the presence of the mediolateral stimulus, it is still worth noticing that head sway was nevertheless reduced by about 20% during both eye movement tasks when compared to the fixation task.

It is important to note that overall head sway across conditions in the anteroposterior direction is about a factor of 2 larger than that of mediolateral head sway (ratio = 2.12). It is therefore possible that the anteroposterior component of balance measured here dominates the 2-D response. As a result, any changes to mediolateral sway across tasks would go unnoticed. In an effort to shed light on this issue, we will now present data separately for each movement axis (i.e. 1-D head sway).

**Fig 6:**
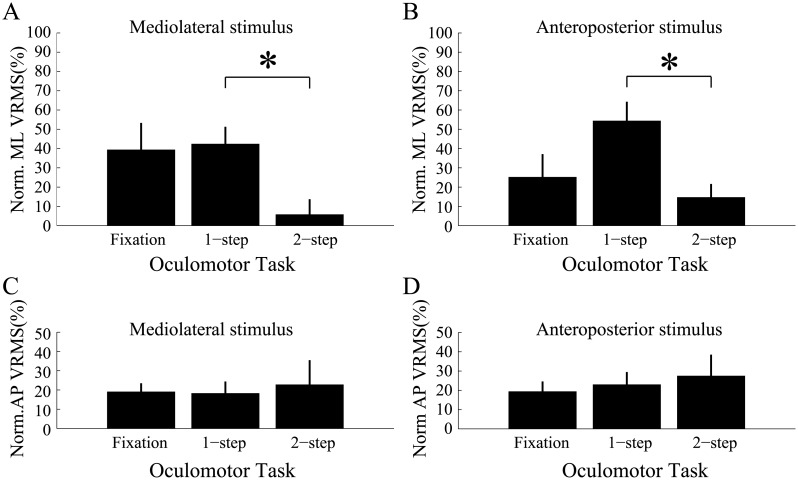
Mean normalized 1-D vRMS as a function of oculomotor task. Checkerboard stimulus moving in the (B,D) anteroposterior and (A,C) mediolateral directions. Mediolateral (A,C) and anteroposterior (B,D) postural measures are presented in separate panels. Data from all subjects and trials was pooled. Error bars = standard error of the mean. * denotes a significant difference (p < 0.05). ML = mediolateral, AP = anteroposterior.

Fig 6 shows for each dynamic visual conditions separately, the normalized mediolateral vRMS (Fig 6A and 6B) and the normalized anteroposterior vRMS (Fig 6C and 6D). It is apparent for both the peripheral mediolateral stimulus (Fig 6A) and the peripheral anteroposterior stimulus (Fig 6B) that mediolateral head sway was clearly reduced during the double-step saccade experiments compared to the single-step saccade experiments. A repeated-measures ANOVA analysis with peripheral stimulus (STIM) and oculomotor task (TASK) as factors confirmed the main effect of the task (TASK: F_(2,22)_ = 3.73, p < 0.05, η_p_^2^ = 0.25) and the absence of an effect of the peripheral stimulus (STIM: F_(1,11)_ = 0.48, p = 0.50). This analysis also revealed a significant interaction (F_(2,22)_ = 5.29, p < 0.05, η_p_^2^ = 0.33). Post-hoc analyses revealed that in the presence of either of the peripheral stimuli less postural instability was found when double-step saccades were performed then when single-step saccades were executed (p < 0.05). With the mediolateral peripheral stimulus, the normalized ML sway during the 2-step task compared to that during the fixation task was clearly reduced (Fig 6A), but this difference failed to reach significance. To explore this further, we conducted a repeated measures ANOVA with only oculomotor task as a factor and found a marginal difference (F_(2,22)_ = 3.20, p = 0.06, η_p_^2^ = 0.23). No statistical differences across oculomotor tasks and stimuli were found for the anteroposterior VRMS (ANOVA, p>0.1) (Fig 6C and 6D).

## Discussion

In this paper we investigated using a new dual-task paradigm whether and how eye movements of different complexity affected balance and whether gaze shift accuracy and precision were compromised in standing subjects following visually-induced postural perturbations. Given the nature of our task, we were particularly interested in knowing whether the two systems studied, the oculomotor and the postural systems, would collaborate to achieve a common goal or whether a trade-off in performance would be observed. As will be discussed below, our results favour the “common goal hypothesis”. We showed that single-step and double-step saccades performed by human subjects from a standing position are precise and accurate. However, for equivalent performance to be observed, “tighter” control of posture was necessary during the more challenging of the two oculomotor tasks, i.e. during the double-step saccades. Contextual modulation of posture was observed in the form of a reduction in head sway specifically along the mediolateral axis. It has been shown previously in sitting human subjects that gaze shift performance did not grossly deteriorate when subjects were asked to perform open-loop sequences of two saccades as opposed to single-step saccades. This is true both for gaze shifts accomplished by eye saccades only [25, 26, 57–61] or through coordinated movements of both the eyes and the head [62]. Here we demonstrate that similar results are also obtained in standing humans whose stance was additionally perturbed using long-duration, wide-field visual stimuli moving sinusoidally either in the anteroposterior or mediolateral direction; in and of themselves these peripheral stimuli are known to increase postural reactivity [56, 63] (see Fig 4, ST vs AP and ML).

### Gaze accuracy is resistant to a variety of perturbation paradigms

The experiments presented here showed that single-step and double-step saccadic eye movements performed by standing healthy adults were accurate and precise, with no differences found across eye movement tasks and no differences found when a dynamic peripheral stimulus was used versus a stable one (Fig 3). As such, our eye movement results are in agreement with those of Stoffregen et al. [1, 4, 5] and Rey et al. [14] who also recorded and analyzed single-step eye movements produced from an upright stance. Both groups found that saccades produced in that context were accurate.

It has been shown that accurate gaze shifts can be produced in a wide variety of contexts and are resistant to different types of perturbations. It is well established that gaze shifts made through coordinated movements of the eyes and head are as accurate as those produced using eye-only movements and as those produced during either passive body rotations or mechanical head-on-body perturbations [64–76]. Moreover, over a range of up to 70 degrees of visual angle, gaze shifts produced using coordinated movements of the eyes, the head, the trunk and the feet are also very accurate [77–79]. Finally, gaze shift endpoint accuracy is also maintained when trajectories are perturbed by either artificially evoking blink movements [80–82] or by electrically stimulating important oculomotor areas such as the superior colliculus [83–87] or the frontal eye field [88]. Here we add to this literature by showing that gaze accuracy is also preserved in standing subjects whose stance was perturbed using wide-field visual stimuli. This is true both when subjects are required to produce single-step and double-step saccades. Taken together, these results demonstrate that goal-directed eye/gaze movements are extremely resistant to an impressive range of perturbation regimes. The importance of maintaining the accuracy of goal-directed movements such as the ones described here despite the great challenges to the system imposed by such perturbations is without a doubt of great value to the organism. It reiterates the important role played by neural and biomechanical compensatory mechanisms in maintaining clear vision as one moves through its environment, especially when faced with perturbations that may prevent the agent from reaching its goal.

The nature of the specific compensatory mechanisms responsible for the maintenance of gaze accuracy will be addressed in future research. We nevertheless speculate that gaze control centers incorporate postural feedback during the remapping process to plan and execute accurate double-step saccades from a standing position. Corollary discharges (CDs) of gaze motor commands are thought to be crucial during the remapping process necessary to generate accurate double-step gaze shifts and we hypothesize that for accurate movements to be produced that CDs are combined with other inputs (vestibular, proprioceptive, postural, visual) in a context-dependent manner through a weighting mechanism. This multisensory integration process insures both that gaze will land on target and that perceptual stability will not be compromised (see [89] for a recent review of similar ideas).

### Reducing mediolateral head sway to facilitate concurrent oculomotor task

Research on the impact of gaze shifts on postural control has shown that body or head or torso sway either decreases [1, 4, 5, 9, 11–18, 20] or remains unchanged [2, 3, 6, 12, 19] when single-step saccades are produced against a static background compared to when subjects maintain fixation on a static central visual stimulus. Our results with a static background agree somewhat with those who found that sway remains unchanged when saccades are produced (Fig 4; ST vs oculomotor tasks). There was however a trend for sway to increase when eye movements were produced, but that result was not significant. This partial discrepancy between our results and those cited above may stem from the fact that most of the previous studies sway using the center of foot pressure (CoP). Those who reported ML head sway like we did either showed that ML head sway decreased [4] during a saccade task (versus a fixation task) or did not change [1]. It would be interesting in future experiments to record the CoP during our task to compare with the current head sway results.

Here we tried to shed light on the interaction between the postural and oculomotor systems using a different approach. We varied the complexity of the oculomotor task and we investigated how the systems adapted to visually-induced postural perturbations. The use of wide-field moving stimuli, as has been shown previously by us using stimuli with dynamics similar to the ones used during our experiments [56, 63], proved adequate to generate increases in 2-D head sway in all three oculomotor tasks (Fig 4). Movement of the peripheral stimulus in both directions (i.e. AP and ML) gave similar results. The major finding of this study with respect to postural control is that visually-induced head sway was reduced specifically in the mediolateral direction and only during the double-step task by about 30% (see Fig 6). No such modulation was found during the other two oculomotor conditions and along the anteroposterior axis. We therefore conclude from these results and those presented in the previous section that for equivalent saccadic performance to be observed when visually-induced postural perturbations were used, a tighter control over ML sway was necessary during the production of sequences of eye movements. It appears that the impact that the whole-field stimulus could have had on eye saccades was efficiently filtered out through axis-specific postural adjustments made along the mediolateral axis.

Though it failed to reach significance, visually-induced 2-D head sway was nevertheless reduced by a good 20% during both single-step and double-step saccades compared to when subjects simply fixated without making saccades (Fig 5). This result was only observed when the mediolateral peripheral stimulus was used to induce head sway. This result complements nicely the 1-D results presented above. The oculomotor tasks required subjects to make much larger saccades in the horizontal plane. The mediolateral stimulus in that context most certainly posed a greater challenge to the oculomotor system than the anteroposterior stimulus.

### The oculomotor and postural systems work together to achieve a common suprapostural goal

At odds with our results is the so-called posture first strategy reported in the literature. This strategy claims that balance should be the primary goal and that a sacrifice in performance should be observed on the secondary task (here eye movements) during dual-task experiments. Our results do not support this hypothesis. We also did not find any clear indications of a trade-off in performance which would lend support to the idea that the two systems studied here were competing for limited processing resources.

It is also be possible that head sway diminished as the oculomotor task increased in complexity simply because overt attentional resources in such instances were redistributed toward the oculomotor task. Withdrawing attention from the postural task allowed the postural system to function more optimally in a more automatized fashion. Though interesting, we do not favour this explanation since decreases in head sway were only found during the double-step task and only in the ML direction. The specificity of this effect cannot easily be predicted from U-shaped models such as the one just mentioned. For a more detailed and recent presentation of those ideas see [90, 91].

Our results are instead in general agreement with the common goal hypothesis and with those of Stoffregen et al. [4] who postulated that: « …postural control can be tuned to support the achievement of suprapostural activities… ». We add that the complexity of the suprapostural task appears to play an important role with regards to this tuning since similar results were not found during single-step saccades. Our results further highlight the importance of analyzing sway separately along the mediolateral and anteroposterior axes since independent control of each axis appears to be possible and often desirable in different contexts.

## Conclusions

Single-step and double-step eye saccades produced from an upright position were shown to be accurate and precise. Moreover we showed that saccade accuracy was not compromised when visually-induced postural perturbations were used to challenge the system. Our data show that a tighter control of posture in the form of reduced mediolateral head sway was necessary in order to produce accurate double-step saccades. Our results therefore showed that postural activity can be modulated in a goal-directed manner to promote the production of accurate sequences of saccades. The new dual-task paradigm designed for this study using visually-induced postural perturbations and oculomotor tasks of varying difficulty allowed us to study the interaction of the oculomotor and postural systems from a different angle and may be prove to be a useful tool for future research.

## Study limitations

One limitation of our study was the small sample size of twelve participants. It will be important in future work to investigate with more subjects whether ML head sway during fixation would also differ significantly from that during single-step saccades (Fig 6B) and double-step saccades (Fig 6A). Despite this limitation, we are confident given the small inter-subject variation observed during our experiments that our results provide a reliable estimate of our outcome measures.

Another limitation of our study may be that 32-second trials may not be adequate to optimize the stability and reliability of a measure such as the vRMS [92, 93]. Recommendations were made that trial duration should be at least 60 seconds. A survey was performed by us of all studies that looked at the effect of eye movement on posture and we found that in the majority of the studies (60%) the duration of a trial was between 22–35 seconds. Only 20% (5/25) of all studies used trial duration longer than 60 seconds and only another 4 studies (16%) used trial duration of about 50 seconds. We are therefore confident that our results will be comparable on methodological grounds to the majority of the studies so far published on the subject. We nevertheless suggest, as others have, that future studies should use a longer durations to avoid confusion.

It may also be argued testing subjects using a fixed task order may be problematic; in the experiments presented here all subjects first performed the double-step saccade task, followed by the single-step saccade task and finally the fixation task. We chose to do so to ascertain that subjects would not memorize the position of the targets before they performed blocks of double-step saccades. We were interested to investigate whether accurate double-step saccades could be performed that required the online remapping of the second target position and to do so we chose to use many initial eye positions, many different sequences of saccades and variable timing to discourage anticipatory and stereotypical behaviours. Given that less head sway was observed during the more complex task and that this task was performed before the other ones, we are confident that task order did not lead to learning effects that could obscure our results.

Finally, though subjects were clearly instructed to place equal emphasis on both tasks performed, we can’t be certain that they did so.

## Future research

It would be interesting in future studies to use the dual-task paradigm presented in this article to investigate other types of eye movements, such as antisaccades, smooth pursuit eye movements and step-pursuit sequences of eye movements. It would also be of interest to adapt our dual-task paradigm to study other behaviours, such as pointing and grasping.

Additionally, a more detailed analysis of the remapping process used during double-step saccades produced from a standing position in order to maintain the accuracy of double-step saccades will also be necessary in future research.

Finally, it would also be pertinent to extend our results through recordings of other body segments such as the torso and to use other measures such as the center of mass (CoM) or the center of foot pressure (CoP).

## Supporting information

S1 File (DOCX)
