# Supplementary material for: Interaction between the oculomotor and postural systems during a dual-task: Compensatory reductions in head sway following visually-induced postural perturbations promote the production of accurate double-step saccades in standing human adults
Source: PLoS One. 2017 Mar 15;12(3):e0173678. doi: 10.1371/journal.pone.0173678 (PMC5351857; doi:10.1371/journal.pone.0173678)
Supplement: S1 File — (DOCX) [file pone.0173678.s001.docx]

**Supporting information**

**S1 file. Head rotation analyses.** Distributions of head direction in yaw were generated and we then tested whether the center of the distributions differed from our calibrated zero position, i.e. straight ahead. We pooled data across trials (separately for each condition) and across subjects. No learning effect was found; none of the regression slopes of mean head direction vs trial number differed from zero as verified with t-tests (All P values >0.05). Care was taken to make sure that no outliers were included in the data set by plotting all data together along with the grand mean. Visual inspection again confirmed that all data points lay within 1 STD of the grand mean.

For none of the conditions were distribution means significantly shifted away from the straight ahead position (t-test, p > 0.05). Additional analyses were performed to determine whether fluctuations in head direction with respect to straight ahead varied across conditions. To do so, we measured the standard deviation over the whole duration of the trial. Two-way repeated-measures ANOVAs revealed no significant effects of the type of checkerboard stimulus used (F_(2,22)_ = 0.09; p = 0.92) and of the oculomotor task performed (F_(2,22)_ = 2.01; p = 0.16). No significant interaction (F_(4,44)_ = 1.02; p = 0.41) was also found. We also investigated whether the absence of head rotation reported above was due to: 1) the effect of pooling trials together and/or 2) of averaging head position over the duration of a whole trial. To perform those analyses, first mean head position samples of 2 seconds were generated every 2 seconds and those data points were fitted using linear regression. We then compared the linear regression slopes across trials for each task separately. Repeated-measures ANOVAs did not reveal any differences in slopes as a function of trial number for any of the conditions (all p values > 0.1). Note also, that regression slopes also did not differ when anteroposterior and mediolateral dynamic trials were pooled separately and compared (Fixation task: F_(1,11)_ = 0.001, p = 0.98; single-step saccade task: F_(1,11)_ = 1.85, p = 0.20; double-step saccade task: F_(1,11)_ = 2.54, p = 0.14). The range of regression slopes (i.e. absolute values) across subjects and tasks was 0.13 ± 0.03. From these analyses, it appears that subjects did not significantly rotate their head during any of the tasks and as such complied with the instructions.
